# Enhancing Classification Performance of Functional Near-Infrared Spectroscopy- Brain–Computer Interface Using Adaptive Estimation of General Linear Model Coefficients

**DOI:** 10.3389/fnbot.2017.00033

**Published:** 2017-07-17

**Authors:** Nauman Khalid Qureshi, Noman Naseer, Farzan Majeed Noori, Hammad Nazeer, Rayyan Azam Khan, Sajid Saleem

**Affiliations:** ^1^Department of Mechatronics Engineering, Air University, Islamabad, Pakistan; ^2^Department of Electrical and Computer Engineering, Institute of Systems and Robotics, University of Coimbra, Coimbra, Portugal; ^3^Faculty of Engineering and Computer Sciences, National University of Modern Languages, Islamabad, Pakistan

**Keywords:** functional near-infrared spectroscopy, brain–computer interface, general linear model, least squares estimation, adaptive estimation, support vector machine

## Abstract

In this paper, a novel methodology for enhanced classification of functional near-infrared spectroscopy (fNIRS) signals utilizable in a two-class [motor imagery (MI) and rest; mental rotation (MR) and rest] brain–computer interface (BCI) is presented. First, fNIRS signals corresponding to MI and MR are acquired from the motor and prefrontal cortex, respectively, afterward, filtered to remove physiological noises. Then, the signals are modeled using the general linear model, the coefficients of which are adaptively estimated using the least squares technique. Subsequently, multiple feature combinations of estimated coefficients were used for classification. The best classification accuracies achieved for five subjects, for MI versus rest are 79.5, 83.7, 82.6, 81.4, and 84.1% whereas those for MR versus rest are 85.5, 85.2, 87.8, 83.7, and 84.8%, respectively, using support vector machine. These results are compared with the best classification accuracies obtained using the conventional hemodynamic response. By means of the proposed methodology, the average classification accuracy obtained was significantly higher (*p* < 0.05). These results serve to demonstrate the feasibility of developing a high-classification-performance fNIRS-BCI.

## Introduction

A brain–computer interface (BCI) system bypasses the peripheral nervous system and provides means of communication for patients suffering from motor disabilities or in a persistent vegetative state using devices, such as robotic arms or other prostheses (Wolpaw et al., 2002). The brain signals are acquired either invasively or non-invasively. Although the quality of brain signals acquired using invasive methods is better than those using non-invasive methods, their acquisition entails extensive surgical risk (Wester et al., 2009). With non-invasive methods, on the other hand, there is no such risk. Non-invasive techniques include electroencephalography (EEG) (Wolpaw et al., 2002; Pfurtscheller et al., 2003; Salvaris and Sepulveda, 2010; Cong et al., 2011, 2015; Jin et al., 2011, 2014, 2015; Choi, 2013; Chen et al., 2015), functional magnetic resonance imaging (fMRI) (Enzinger et al., 2008; Sorger et al., 2009), and functional near-infrared spectroscopy (fNIRS) (Ferrari et al., 1985; Kato et al., 1993; Coyle et al., 2004, 2007; Naito et al., 2007; Naseer and Hong, 2013; Naseer et al., 2014; Noori et al., 2017). Over the course of the past decade, fNIRS-based BCI systems have been the focus of considerable research interest and discussion due to their portability, affordable cost and better temporal resolution relative to fMRI. Moreover, compared with the EEG system, they offer better spatial resolution and a superior signal-to-noise ratio (Hu et al., 2012; Hong et al., 2014). In general, fNIRS has evolved into a neuroimaging technique that has contributed to ground-breaking advances in the understanding of human brain functionality (Irani et al., 2007; Aqil et al., 2012b; Ferrari and Quaresima, 2012; Hong and Nguyen, 2014; Hong and Naseer, 2016; Hong and Santosa, 2016). fNIRS utilizes near-infrared (NI) light within the 650–1000 nm wavelength range to measure changes in the concentrations of oxygenated and deoxygenated hemoglobin [Δ*c*_HbO_(*t*) and Δ*c*_HbR_(*t*)] according to the modified Beer–Lamberts Law (Delpy et al., 1988; Villringer et al., 1993; Hoshi et al., 1994; Hoshi and Tamura, 1997). Since the introduction of the principle of NI spectroscopy by Jobsis (1977), fNIRS has been used effectively for functional and structural brain imaging as well as for BCI purposes (Naseer and Hong, 2015a,b; Nguyen et al., 2016; Zafar and Hong, 2017). The first step in fNIRS-BCI is to acquire signals from a suitable mental task. Over the past decade, the mental tasks used by fNIRS-BCI researchers have been motor imagery (MI), mental rotation (MR), mental arithmetic, music imagery, and letter padding (Zhang et al., 2011a; Ayaz et al., 2013, 2014; Khan et al., 2014; Khan and Hong, 2015). In this study, we used right-hand MI and MR as the brain activity. Generation of control commands for fNIRS-based BCI systems proceeds according to the following conventional steps: first, acquisition of the desired signals; second, removal of motion artifacts and physiological noises; third, extraction of significant information (features), usually from the hemodynamic signals’ physical properties; fourth and finally, classification of the extracted features preparatory to generation of the desired control commands. Researchers have devoted considerable efforts to the improvement and enhancement of classification accuracies for fNIRS-BCI, specifically by use of different features and classifiers (Ayaz et al., 2013, 2014; Naseer and Hong, 2013; Naseer et al., 2014; Noori et al., 2016; Qureshi et al., 2016; Khan and Hong, 2017). In this paper, we propose that features be extracted from the estimated coefficients of the general linear model (GLM).

The GLM methodology was first employed by Abdelnour and Huppert (2009) in a fNIRS-based BCI study. Since that time, multiple GLM-based fNIRS studies have been performed for noise removal and brain mapping (Hu et al., 2010; Zhang et al., 2011b, 2012; Aqil et al., 2012a; Kamran and Hong, 2013). Abdelnour and Huppert (2009) have proposed the use of filter coefficients obtained by Kalman filtering as the features for classification. They assumed that different brain activities will produce different filter coefficients, using which different signals can be classified. Similarly, recursive least square estimation (Aqil et al., 2012a), and wavelet transform (Khoa and Nakagawa, 2008; Abibullaev et al., 2011; Abibullaev and An, 2012) have also been used for brain mapping using GLM. In this study, GLM is used with least square to estimate filter coefficient values. Afterward these values are used to extract features. To the best of our knowledge, this is the first work that uses filter coefficient values to extract statistical features that can be used for classification. In the proposed methodology, signals are acquired from the left motor cortex of the brain for right-hand MI (clenching of the right hand) and rest tasks, whereas MR (rotation of rectangular box) and rest signals are acquired from the prefrontal cortex; these signals are filtered to remove physiological noises and the GLM coefficients are extracted using the least squares estimation (LSE) technique; the feature values of these coefficients are then fed to support vector machine (SVM) for classification. The motivation of using GLM-based features for fNIRS data came from Abdelnour and Huppert (2009). They showed promising results using beta (β) values extracted from GLM as features. In this study, authors have used GLM with least square to estimate β values. Afterward β values are used to extract features in order to calculate classification accuracies.

## Materials and Methods

### Experimental Procedure

#### Subjects

A total of 10 subjects participated in the experiments. Five subjects performed MI (right-hand clenching) versus rest, whereas the other five performed MR (rotation of rectangular box) versus rest. The reason for introducing two different experiments was to establish generalization of the proposed methodology. The subjects were each seated on a comfortable chair in front of a display screen and asked to restrict their body movements as much as possible during the experiment. Verbal consent was obtained from all of the subjects after explaining the experimental paradigm in detail. The subjects had little or no previous experience of fNIRS recording. This work was approved by the Institutional Review Board of Pusan National University. All experiments were conducted in accordance with the ethical standards encoded in the latest Declaration of Helsinki. The complete details of the baseline system (conventional methodology) can be found in Naseer et al. (2016a,b).

#### Motor Imagery

The first 20 s was the rest period, required in order to set up the baseline condition; it was followed by 20 s of a right-hand MI task (clenching of the right hand), followed by another 20 s rest period that allowed the signals to return to their baseline values before the start of the next trial. This pattern was repeated 11 times; the total duration of experiment for each subject, therefore, was 440 s. During the MI task, the subjects were asked to imagine clenching of their right hand with a self-paced frequency of around 1 Hz; during the rest period, they were asked to relax.

#### Mental Rotation

Similar to MI task in MR task the first 20 s was the rest period, required in order to set up the baseline condition; it was followed by 10 s of object rotation (rotation of rectangular box), followed by another 20 s rest period that allowed the signals to return to their baseline values before the start of the next trial. This pattern was repeated 10 times; the total duration of experiment for each subject, therefore, was 330 s. During the MR task, the subjects were asked to imagine rotation of a rectangular box; during the rest period, they were asked to relax.

#### Signal Acquisition

**Figure 1:**
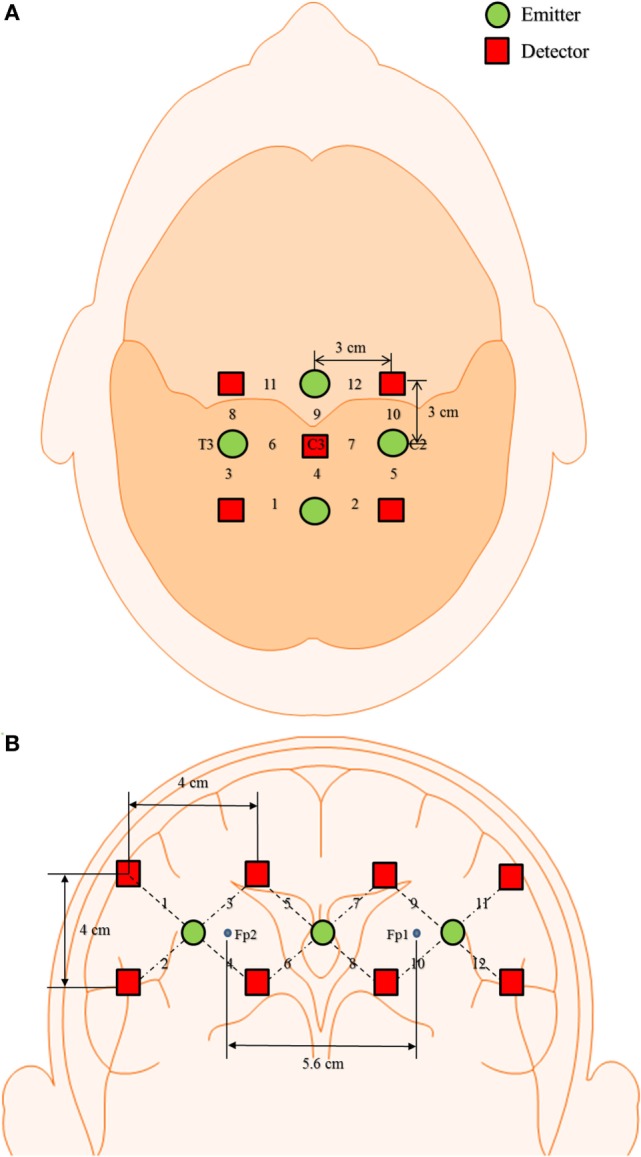
Optode placement and channel location. **(A)** 12-channel with 4 detectors and 5 emitters on the left motor cortex and **(B)** 12-channel with 8 detectors and 3 emitters on the prefrontal cortex of brain region.

In order to acquire fNIRS signals from the right motor cortex of the brain, a multi-channel continuous-wave imaging system (dynamic near-infrared optical tomography; two wavelengths: 760 and 830 nm; NIRx Medical Technologies, NY, USA) with a sampling rate of 1.81 Hz was employed. The acquired signals’ light intensities were first converted to Δ*c*_HbO_(*t*) and Δ*c*_HbR_(*t*) using the modified Beer–Lamberts Law

(1) $$\left[\begin{array}{c} \Delta c_{\text{HbO}}\left(t\right)\\ \Delta c_{\text{HbR}}\left(t\right) \end{array}\right]=\frac{1}{l\times d}{\left[\begin{array}{cc} \alpha_{\text{HbO}}\left(\lambda_{1}\right) & \alpha_{\text{HbR}}\left(\lambda_{1}\right)\\ \alpha_{\text{HbO}}\left(\lambda_{2}\right) & \alpha_{\text{HbR}}\left(\lambda_{2}\right) \end{array}\right]}^{-1}\left[\begin{array}{c} \Delta A\left(t,\;\lambda_{1}\right)\\ \Delta A\left(t,\;\lambda_{2}\right) \end{array}\right]$$

where Δ*A*(*t*; λ*_j_*) (*j* = 1, 2) is the unit-less absorbance (optical density) variation of a light emitter of wavelength λ*_j_*, *a*_HbX_(λ*_j_*) is the extinction coefficient of HbX (HbO and HbR) in μM^−1^mm^−1^, *d* is the unit-less differential path length factor, and *l* is the distance (in millimeters) between the emitter and detector. As shown in Figure 1A, four emitters and five detectors were positioned over the left motor cortex of the brain for right-hand MI task. Figure 1B shows eight emitters and three detectors placed on prefrontal cortex of the brain region in order to acquire signals for MR, the distance between each emitter–detector pair was of 3 cm. This emitter–detector distance is in accordance with the literature (McCormick et al., 1992; Gratton et al., 2006). In order to remove physiological noises (heartbeat, respiration) from the obtained signals, the Butterworth filter of order four was used with a cut-off frequency of 0.6 Hz; for removal of low-oscillation Mayer waves, a high-pass filter with a cut-off frequency of 0.01 Hz was used (Naseer and Hong, 2015a,b).

### Methodology

#### General Linear Model

The GLM has been very widely utilized by researchers of fNIRS-BCI systems in order to identify brain-activation patterns for multiple cognitive tasks (Abdelnour and Huppert, 2009; Hu et al., 2010; Zhang et al., 2011b, 2012; Aqil et al., 2012a; Kamran and Hong, 2013). The GLM-based methods were developed initially for fMRI-based functional brain mapping. To analyze fMRI data, GLM methodology has been developed to explain the timeline blood oxygenation level dependent signal. Currently, they are frequently used in fNIRS studies. The GLM defines measured data in the form of a linear combination of several variables and an error term. The observation of hemodynamic changes can be expressed as

(2) y=Gβ+e

where the *y* vector represents the measured data (in fNIRS, the vector is the observed time-series of the hemodynamic response), *G* is the design matrix obtained by convolving the canonical hemodynamic response with the experimental box-car function (Ye et al., 2009), β is the set of coefficients for the functional response that we want to estimate, and *e* is the error term. The vital part of the model function of a GLM is the box-car function, which reflects the temporal structures of the experimental paradigm and is convolved with the canonical hemodynamic response function (Ye et al., 2009). As physiological noises had already been removed using the Butterworth filter, only one explanatory variable (the design matrix) was used to extract the β values.

#### Least Squares Estimation

Least squares estimation is used to estimate the β values from the GLM. The time-course values predicted by the model are obtained by linear combination of the predictors

(3) $$\hat{y}=G\beta$$

In order to achieve a good fit, the β values should be close to the predicted values that are as close as possible to the measured values *y*. Thus, the system of equations should be rearranged as

(4) e=y−Gβ

(5) $$e=y-\hat{y}$$

Although the GLM methodology does not estimate β values, it can be applied to minimize the sum of squared error values by using

(6) $$e^{\prime }e={\left(y-G\beta \right)}^{\prime }\left(y-G\beta \right)\to \text{min}$$

where *e′e* shows the vector notation for the sum of squares. Utilizing LSE, the β weights minimizing the square error values are obtained by

(7) $$\beta ={\left(G^{-1}G\right)}^{-1}G^{\prime }y$$

**Figure 2:**
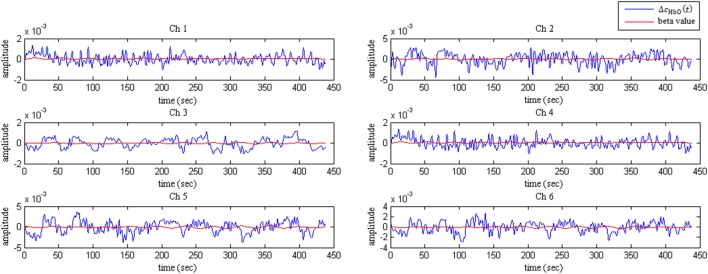
Δ*c*_HbO_(*t*) signals and their corresponding adaptively estimated β values for subject 5.

The resulting matrix (*G*^−1^*G*)^−1^ plays an important role in the calculation of the β values. The remaining term on the right side, *G′y*, evaluates a vector containing as many elements as predictors. Figure 2 plots the Δ*c*_HbO_(*t*) signals for the MI task and rest period with their corresponding adaptively estimated β values for subject 5.

#### Feature Extraction and Classification

In this study, the statistical properties of the β values were used as the features. Signal peak (SP), signal skewness (SSk), signal mean (SM), signal variance (SV), signal kurtosis (SK), and signal slope (SS) were extracted from the β values obtained by LSE. The SSk values were determined by measuring the asymmetry of the signal values around the mean relative to a normal distribution:

(8) $$\text{skewness}\left(Y\right)=E\left[{\left(\frac{Y-\mu }{\sigma }\right)}^{3}\right]$$

where *E* is the expected value of *Y* and σ is the SD of *Y*

(9) $$\text{mean}=\frac{1}{N}\sum_{i=1}^{N}Y_{i}$$

where *N* is the number of observations and *Y_i_* represents the β values. The variance is calculated as follow:

(10) $$\text{variance}\left(Y\right)=\frac{\sum {\left(Y-\mu \right)}^{2}}{N}$$

where μ is the mean value of *Y*. The kurtosis is computed as follows:

(11) $$\text{kurtosis}\left(Y\right)=E\left[{\left(\frac{Y-\mu }{\sigma }\right)}^{4}\right]$$

The SS is calculated using the *polyfit* function in MATLAB^®^. The SP values, which measure the peaks of signals, were determined using MATLAB^®^*max* function. These features were calculated across all 12 channels for the MI and MR. All of the feature values were scaled between 0 and 1 using the equation

(12) $$x^{\prime }=\frac{x-\text{min}\left(x\right)}{\text{max}\left(x\right)-\text{min}\left(x\right)}$$

where *x* ∈ *R^n^* represents the original feature values, *x*′ denotes the rescaled feature values between 0 and 1, max(*x*) is the largest value, and min(*x*) is the smallest value. After extracting the features from the β values, SVM was used to classify the MI and MR tasks (Naseer et al., 2016b). SVM maximizes the margins between classes by creating hyperplanes that minimize the cost function

$$\begin{array}{l} \text{Minimize }\frac{1}{2}{||w||}^{2}+C\sum_{i=1}^{n}\xi_{i}\\ \text{Subject to }z_{i}\;\left(w^{T}x_{i}+b\right)\geq 1-\xi_{i},\;\xi_{i}\geq 0 \end{array}$$

where *w^T^*, *x_i_* ∈ *R*^2^ and b ∈ *R*^1^, ||*w*||^2^ = *w*^T^*w*, *C* is the trade-off parameter between the error and the margin, ξ*_i_* is the measure of the training data, and *z_i_* is the class label for the *i*-th sample. The most significant advantage of SVM is that it can be used as a linear as well as a non-linear classifier; in fact, in this study, a third-degree polynomial kernel function was used with *C* = 0.5. Ten-fold cross-validation was utilized to extract the classification accuracies for the MI and MR tasks versus rest periods. Moreover, in order to measure classification performance, recall and precision were calculated for both paradigms as follows:

(13) $$\text{Recall}=\frac{\text{TP}}{\text{TP+FN}}$$

(14) $$\text{Precision}=\frac{\text{TP}}{\text{TP+FP}}$$

where TP, FP, and FN denote true positive, false positive, and false negative, respectively. These values were calculated from confusion matrix (Fawcett, 2006).

## Results

**Figure 3:**
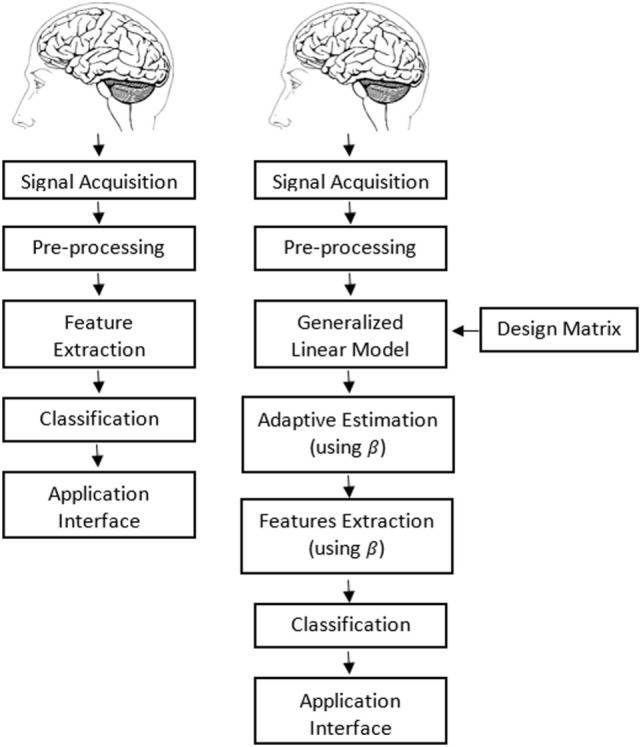
Schematic of **(A)** proposed and **(B)** conventional methodology.

Multiple feature combinations were used in order to extract significant classification accuracies for proposed and conventional methodologies. The classification accuracies obtained for the five subjects using the proposed method for MI versus rest were 79.5, 83.7, 82.6, 81.4, and 84.1% using SM and SSk, whereas those for MR versus rest were and 85.5, 85.2, 87.8, 83.7, and 84.8% using SP and SSk. To establish the superiority of the proposed method over the previous methods, the classification accuracies using the conventional hemodynamic response feature also were calculated. Figures 3A,B provides a schematic of the conventional and proposed methodology for fNIRS-based BCI study. Furthermore, the classification accuracies obtained for the five subjects using the conventional method for MI versus rest were 60.4, 78.9, 70.4, 68.9, and 54.4% using SM and SP, whereas those for MR versus rest were and 66.7, 73.0, 72.2, 68.5, and 63.3% using SM and SP. Tables 1 and 2 list the classification accuracies, precisions, and recalls of all subjects using the proposed methodology and the conventional method, for all possible two-feature combinations for MI versus rest task, respectively. Tables 3 and 4 list the classification accuracies, precisions, and recalls of all subjects using the proposed methodology and the conventional method, for all possible two-feature combinations for MR versus rest task, respectively. The results show that in MI task the optimal feature combinations that yielded best classification accuracies were “SM and SSk” and “SM and SP” for beta values and conventional hemodynamic response, respectively. In MR task, the optimal feature-combination that yielded best classification accuracies were “SP and SSk” and “SM and SP” for beta values and conventional hemodynamic response, respectively. In order to ensure that the data are normally distributed Kolmogorov–Smirnov method was applied, the significant value was found to be greater than 0.05 which shows normal distribution of the data. These high classification accuracies of the proposed method relative to the conventional method were statistically verified by a statistical significance test (the Student’s *t*-test): the *p*-values obtained by performing *t*-test on the subject-wise accuracy scores was less than 0.05, which confirmed the statistical significance of the proposed methodology’s superior performance for both tasks.

**Table 1:** Classification performances of proposed methodology for motor imagery versus rest task across all feature combinations.

Feature combinations	S1			S2			S3			S4			S5		
	Accuracy	Precision	Recall	Accuracy	Precision	Recall	Accuracy	Precision	Recall	Accuracy	Precision	Recall	Accuracy	Precision	Recall
Signal peak (SP) and signal skewness (SSk)	76.3	73.6	78.8	82.3	78.1	91.9	73.9	70.3	85.6	84.9	87.2	82.3	89.9	90.9	92.9
Signal mean (SM) and SSk	79.5	78.0	74.0	83.7	91.2	82.3	82.6	85.7	75.8	81.4	89.5	71.1	84.1	87.1	75.9
Signal slope (SS) and SSk	73.6	75.3	70.4	63.4	72.3	36.8	69.3	65.0	83.8	79.6	71.8	97.5	87.9	93.4	81.6
Signal kurtosis (SK) and SSk	71.1	80.3	56.0	55.4	60.3	31.8	66.4	73.1	52.0	80.5	86.0	72.9	82.9	83.7	81.6
Signal variance (SV) and SSk	78.5	74.1	87.7	87.4	88.5	85.9	60.6	61.0	59.2	74.4	70.1	84.8	90.4	89.2	92.1
SP and SK	76.4	73.5	82.3	80.1	74.1	92.8	71.3	76.8	61.0	81.4	86.6	74.4	84.8	77.7	97.8
SM and SK	73.5	71.8	77.3	70.4	74.7	61.7	74.5	84.0	60.6	79.1	85.2	70.4	79.1	72.3	94.2
SS and SK	73.6	74.3	72.2	66.1	77.6	45.1	70.6	82.4	52.3	77.1	69.5	96.4	89.9	92.0	87.4
SV and SK	76.4	75.0	79.1	85.7	90.2	80.1	65.2	70.6	52.0	67.3	70.9	58.8	81.9	82.2	81.6
SM and SP	74.4	74.4	74.4	84.7	79.4	93.5	70.4	80.2	54.2	83.4	86.9	78.7	91.5	85.5	98.7
SM and SV	74.7	72.9	78.7	86.3	86.5	85.9	72.2	88.7	50.9	78.7	81.9	73.6	76.7	82.5	67.9
SS and SP	72.4	73.5	70.0	82.1	77.5	90.6	70.6	73.4	64.6	81.9	77.2	90.6	89.0	95.4	81.9
SS and SV	70.4	72.2	66.4	86.6	92.1	80.1	65.9	65.4	67.5	78.5	72.4	92.1	84.7	94.0	74.0
SV and SP	76.2	72.6	84.1	84.3	85.2	83.0	66.4	66.9	65.0	82.3	81.2	84.1	85.4	85.0	85.9
SM and SS	73.5	77.1	66.8	67.5	70.0	61.4	68.6	66.8	74.0	80.0	72.0	98.2	88.1	87.8	88.4

**Table 2:** Classification performances of conventional methodology for motor imagery versus rest task across all feature combinations.

Feature combinations	S1			S2			S3			S4			S5		
	Accuracy	Precision	Recall	Accuracy	Precision	Recall	Accuracy	Precision	Recall	Accuracy	Precision	Recall	Accuracy	Precision	Recall
Signal peak (SP) and signal skewness (SSk)	49.9	48.7	58.1	54.1	59.3	56.8	52.2	54.2	53.5	53.2	51.9	71.7	48.7	47.9	43.9
Signal mean (SM) and SSk	57.0	52.3	28.8	64.8	64.1	58.1	65.9	63.7	59.6	69.3	63.2	74.7	62.2	59.8	71.7
Signal slope (SS) and SSk	55.2	51.7	30.8	53.7	51.7	49.7	64.4	65.2	61.4	52.2	50.7	57.6	53.7	51.2	49.7
Signal kurtosis (SK) and SSk	40.4	52.2	36.4	55.6	54.3	61.6	60.7	56.7	51.0	61.9	57.6	34.8	43.0	41.7	36.5
Signal variance (SV) and SSk	53.3	51.3	39.4	58.5	57.6	54.5	57.0	53.4	71.7	61.5	58.1	41.9	61.9	60.4	55.1
SP and SK	47.8	46.9	43.4	63.7	62.1	60.6	64.8	63.7	53.4	77.0	73.4	66.7	40.7	37.1	32.5
SM and SK	54.8	52.5	32.3	71.9	69.8	64.5	63.0	62.2	58.6	71.9	68.3	59.1	54.8	52.7	49.1
SS and SK	58.9	56.7	48.9	45.2	44.2	21.2	60.7	56.3	55.2	53.0	52.4	32.8	51.1	48.3	43.1
SV and SK	53.7	53.4	32.7	57.0	52.3	22.2	60.4	57.5	54.8	65.2	63.1	57.3	62.2	59.7	63.4
SM and SP	60.4	58.7	64.1	78.9	75.6	69.8	70.4	69.1	65.7	68.9	67.5	59.8	54.4	54.2	49.7
SM and SV	59.6	57.9	56.3	71.1	68.7	63.5	73.0	72.5	69.1	73.0	71.4	68.3	51.9	48.7	46.5
SS and SP	69.6	67.4	58.6	55.9	50.9	57.1	65.2	64.1	59.7	70.0	67.7	65.4	58.1	57.3	63.1
SS and SV	45.6	42.7	20.7	61.1	59.3	53.4	62.2	58.3	55.9	53.7	51.3	27.3	56.3	55.1	46.9
SV and SP	67.4	65.1	59.8	69.6	67.4	56.1	68.1	68.7	61.2	69.6	69.7	55.8	54.1	63.7	59.3
SM and SS	60.4	59.5	33.3	64.8	63.5	59.7	64.1	62.3	58.7	65.6	63.2	59.1	64.8	61.9	58.2

**Table 3:** Classification performances of proposed methodology for mental rotation versus rest task across all feature combinations.

Feature combinations	S1			S2			S3			S4			S5		
	Accuracy	Precision	Recall	Accuracy	Precision	Recall	Accuracy	Precision	Recall	Accuracy	Precision	Recall	Accuracy	Precision	Recall
Signal peak (SP) and signal skewness (SSk)	85.5	91.3	87.8	85.2	82.8	93.9	87.8	97.3	83.3	83.7	77.7	97.2	84.8	85.0	91.6
Signal mean (SM) and SSk	84.1	89.4	87.0	77.0	85.0	81.4	77.0	83.3	96.2	82.4	96.1	82.4	88.5	96.7	87.4
Signal slope (SS) and SSk	82.6	78.9	94.0	80.7	95.6	79.6	71.5	77.8	95.9	80.0	96.1	78.6	78.1	81.7	85.0
Signal kurtosis (SK) and SSk	77.0	76.7	87.3	80.0	91.7	80.9	65.4	85.6	87.2	77.8	87.2	80.9	77.0	72.2	91.5
Signal variance (SV) and SSk	85.6	90.6	88.1	72.2	68.9	86.7	62.9	68.3	88.3	78.5	88.3	81.1	75.2	70.6	90.1
SP and SK	82.6	96.7	80.9	72.2	62.2	94.1	68.4	93.3	70.6	76.7	70.6	92.7	80.4	85.0	85.5
SM and SK	80.0	90.6	81.5	73.0	67.8	89.1	76.0	95.0	70.6	74.4	70.6	88.8	72.6	74.4	82.7
SS and SK	83.3	92.8	83.9	69.3	65.6	84.9	74.0	83.3	78.3	75.2	78.3	83.4	81.5	79.4	91.7
SV and SK	83.3	92.7	84.6	53.0	37.2	82.7	60.9	83.9	78.9	74.4	78.9	82.1	64.1	50.6	91.9
SM and SP	75.6	91.1	76.6	72.2	62.2	94.1	70.5	72.2	51.1	63.7	51.1	90.2	80.7	84.4	86.4
SM and SV	80.0	91.7	80.9	66.7	50.6	98.9	70.5	61.1	64.4	71.1	64.4	89.2	79.6	82.2	86.5
SS and SP	81.5	76.7	94.5	75.9	61.1	96.7	68.4	66.7	58.3	67.8	58.3	89.7	87.4	90.6	90.6
SS and SV	79.6	73.9	94.3	73.0	74.4	83.2	65.4	44.4	60.0	67.4	60.0	87.1	83.3	93.9	83.3
SV and SP	74.1	75.0	84.4	69.6	62.8	88.3	67.4	57.8	52.2	65.2	52.2	92.2	79.6	75.0	93.1
SM and SS	75.6	66.7	95.2	80.4	77.2	92.1	68.4	71.7	54.4	66.7	54.4	92.5	80.7	82.2	88.1

**Table 4:** Classification performances with proposed and conventional methodologies for mental rotation versus rest task.

Feature combinations	S1			S2			S3			S4			S5		
	Accuracy	Precision	Recall	Accuracy	Precision	Recall	Accuracy	Precision	Recall	Accuracy	Precision	Recall	Accuracy	Precision	Recall
Signal peak (SP) and signal skewness (SSk)	56.3	76.9	55.6	62.2	74.8	69.4	58.9	74.2	57.8	61.1	73.6	63.6	58.2	72.9	66.2
Signal mean (SM) and SSk	57.8	50.0	78.9	64.4	76.1	72.1	65.2	82.8	70.3	64.1	74.4	72.4	57.8	54.4	75.4
Signal slope (SS) and SSk	61.5	61.1	76.4	47.8	32.8	74.7	65.2	67.2	77.6	48.9	40.0	70.6	53.0	41.1	77.9
Signal kurtosis (SK) and SSk	56.7	45.0	81.8	61.1	73.9	69.6	51.9	43.9	73.1	58.5	71.1	68.1	40.7	22.2	66.7
Signal variance (SV) and SSk	55.9	38.3	89.6	70.0	66.7	85.1	57.8	48.9	80.0	50.4	43.3	70.9	63.0	70.0	73.3
SP and SK	54.4	41.1	81.3	62.6	77.8	69.7	48.1	29.4	80.3	70.7	79.4	77.3	56.7	72.8	65.8
SM and SK	58.1	55.0	75.6	60.4	71.1	69.9	70.7	94.4	71.1	73.7	88.9	75.8	44.8	26.1	74.6
SS and SK	53.0	34.4	87.3	44.8	21.7	83.0	66.3	73.3	75.4	50.4	35.0	78.8	54.1	51.1	71.9
SV and SK	54.4	33.3	95.2	60.0	45.0	90.0	60.7	64.4	73.4	59.6	66.1	71.3	57.4	58.9	72.1
SM and SP	66.7	72.2	76.5	73.0	79.4	79.9	72.2	92.2	73.1	68.5	78.3	75.4	63.3	83.9	68.3
SM and SV	57.4	41.1	89.2	72.2	72.2	83.9	67.4	85.6	71.3	67.4	76.7	75.0	61.9	81.1	67.9
SS and SP	70.7	72.2	81.8	60.7	50.0	84.9	61.9	60.6	77.3	62.6	63.9	76.2	56.7	62.2	69.6
SS and SV	47.8	24.4	89.8	65.6	56.7	87.2	65.9	67.2	78.6	56.7	60.6	70.3	61.9	72.8	70.8
SV and SP	64.1	50.0	92.8	69.6	81.7	75.0	56.7	58.9	71.1	69.6	74.4	78.8	59.3	66.1	70.8
SM and SS	58.5	46.7	84.0	64.8	57.2	85.1	61.1	56.7	79.1	71.1	82.2	76.3	72.6	95.0	72.5

## Discussion

In previous studies, researchers have focused their efforts on enhancing the classification performance of multiple mental tasks in order to generate commands effective for control of external devices or for communication with patients suffering from amyotrophic lateral sclerosis, locked in syndrome, or other physical disabilities. However, distinct BCI signals for a specific mental task were unsuitable for classification, even when using current advanced methods. Previously, Tai and Chau (2009); Khan and Hong (2015); Naseer et al. (2016a); Naseer et al. (2016b) have used features extracted directly from hemodynamic response in order to acquire classification accuracies. In this study, a novel methodology that proceeds by adaptive estimation of GLM coefficients and extraction of the classification performances of MI versus rest and MR versus rest task were developed and evaluated. The results indicated enhanced classification performance as compared with a conventional hemodynamic-response-based fNIRS-BCI. Moreover, the proposed methodology can enhance classification performance if a user is not able to generate distinct brain signals for a specific mental task. The GLM methodology has been frequently employed to analyze time-series fMRI data: Abdelnour and Huppert (2009) first used the GLM in an fNIRS study in order to minimize physiological noises; soon thereafter, Hu et al. (2010) developed a novel online data analysis scheme using the GLM and Kalman estimator to reduce physiological noises for finger-tapping experiments; Aqil et al. (2012a) presented an online brain-imaging framework for finger-tapping tasks using GLM and a recursive least squares estimation method; Zhang et al. (2011b, 2012) tested multiple recursive algorithms for removal of physiological noises and, thereby, extraction of better neuron-related concentration changes in observed fNIRS data. All of these studies used the GLM for the removal of physiological noises and demonstrated brain-activation mapping for multiple cognitive tasks. However, the GLM coefficients, as estimated using LSE, have not been used as features for classification. The difference in classification accuracies is possible since in the conventional method we use statistical features obtained directly from HbO signals; whereas in the proposed method, we use statistical features obtained from β values. Multiple feature combinations have been used in order to determine optimal feature combination, which yields best classification accuracies, using proposed and conventional methodologies for both mental tasks. It was found that in MI task the optimal feature-combinations that yielded best classification accuracies were “SM and SSk” and “SM and SP” for beta values and conventional hemodynamic response, respectively. In MR task, the optimal feature-combination that yielded best classification accuracy were “SP and SSk” and “SM and SP” for beta values and conventional hemodynamic response, respectively. The proposed method has shown improved overall classification accuracies as compared to conventional methodology. This study showed that there is a significant difference between the classification accuracies of the proposed and conventional methodologies: the result is improved by an average of 18.4% for MI versus rest and 16.7% for MR versus rest using the proposed method. Moreover, it was found that features extracted from proposed methodology are statistically significant from conventional methodology for both paradigms.

It should be noted that in Naseer et al. (2016a), the best two- and three-feature combinations yielded accuracies of more than 90% for a seemingly very similar classification task. In this work, the accuracies obtained are in the range of 70%. These differences in the accuracies might be attributed to different recording conditions and different mental tasks. It is observed that the signal quality in mental arithmetic tasks is better as compared to MI and MR tasks. This could be attributed to user training as well. The subjects used in Naseer et al. (2016a) were regular fNIRS-based BCI users. All subjects in this paper had little or no experience of fNIRS recording/BCI training. The effect of using current method on the data from Naseer et al. (2016a) can be evaluated in future works.

This study has some limitations. The first is that only six features were used for classification. The combination of several other statistical features acquired from β values also should be utilized as features, as, thereby, classification performance could be further enhanced. The second limitation is that only SVM was used as the classifier. The positive effects of several other classifiers, however, have been seen. As shown in Naseer et al. (2016b), classification accuracies acquired using artificial neural networks (ANNs) are better than those acquired using SVM and, therefore, ANN’s can be considered for classification in future studies. The third limitation is that the proposed methodology is complex as compared to conventional method since an extra step of calculating general linear model coefficients is involved. This will increase the computational cost as well. The fourth limitation of this study is the fact that only two mental tasks for each paradigm were considered, which restricts this study to a two-class BCI problem. Certainly in any case, it can be upgraded to a multi-class BCI problem in a further study.

In conclusion, we present a novel methodology for enhanced classification accuracy of two-class fNIRS-based BCI. The hemodynamic signals of five subjects were modeled using the GLM and the beta values estimated by LSE were used to extract the features for classification. The classification accuracies obtained using the proposed methodology were significantly higher than those obtained using conventional hemodynamic-response-based features. These results indeed show enhanced classification performance relative to the conventional methodology and represent a step forward in the important task of making fNIRS-based BCIs more accurate and reliable.

## Ethics Statement

This work was approved by the Institutional Review Board of Pusan National University. All experiments were conducted in accordance with the ethical standards encoded in the latest Declaration of Helsinki.

## Author Contributions

NQ conceived this study and was involved in the experiments, data processing, and writing of the manuscript. FN was involved in the experiments and data analysis. HN and RK were involved in data analysis, rechecking of results, and revision. SS helped in revision of the manuscript. NN was involved in the writing of the manuscript and supervised the research.

## Conflict of Interest Statement

The authors declare that the research was conducted in the absence of any commercial or financial relationships that could be construed as a potential conflict of interest. The reviewer, RC, and handling editor declared their shared affiliation, and the handling editor states that the process nevertheless met the standards of a fair and objective review.
